# Enhancing Transsectoral Interdisciplinary Patient-Centered Care for Patients With Rare Cancers: Protocol for a Mixed Methods Process Evaluation

**DOI:** 10.2196/49731

**Published:** 2023-10-12

**Authors:** Jana Hinneburg, Sandro Zacher, Birte Berger-Höger, Karin Berger-Thürmel, Vanessa Kratzer, Anke Steckelberg, Julia Lühnen, TARGET Group

**Affiliations:** 1 Institute for Health and Nursing Science Medical Faculty Martin Luther University Halle-Wittenberg Halle (Saale) Germany; 2 Institute for Public Health and Nursing Research Faculty of Human and Health Sciences University of Bremen Bremen Germany; 3 Department of Medicine III LMU University Hospital LMU Munich Munich Germany; 4 Comprehensive Cancer Center LMU University Hospital LMU Munich Munich Germany; 5 Charité – Universitätsmedizin Berlin, corporate member of Freie Universität Berlin and Humboldt Universität zu Berlin Institute of Clinical Nursing Science Berlin Germany; 6 see Acknowledgments

**Keywords:** process evaluation, study protocol, logic model, complex intervention, coordination of care, rare cancer, mobile phone

## Abstract

**Background:**

Rare cancers account for approximately 24% of all new cancers. The category of rare tumor diseases includes almost 200 different entities. In particular, the treatment of patients with extensive care needs requires cooperation between service providers, both between sectors (outpatient and inpatient) and within sectors (eg, between different medical disciplines). The treatment pathway is associated with a high need for coordination and information sharing between providers. When crossing sectoral boundaries in the German health care system, interface problems between the outpatient and inpatient sectors can lead to gaps in care delivery. The multicomponent program *Trans-sectoral Personalised Care Concept for Patients with Rare Cancers* aims to optimize transsectoral cooperation and coordination of care to enhance patient involvement and the medical care coordination of patients with rare cancers.

**Objective:**

This process evaluation will contribute to answering questions about intervention fidelity and the implementation of transsectoral communication, identifying and describing the intended and nonintended effects of the intervention, and exploring the barriers to and facilitators of the implementation.

**Methods:**

We will include patients who participate in the intervention phase; all persons and staff involved in the development and implementation of the intervention (Onco Coach, psychologists, physicians on the contact platform, IT staff, and staff of the Bavarian Association of Statutory Health Insurance Physicians); physicians from the Ludwig-Maximilians-University Hospital Munich and the hospital of the Technical University Munich who are involved in the treatment of patients during the course of the project; and participating office–based hematologists and oncologists. Data collection will be conducted at the beginning, during, and at the end of the intervention using mixed methods. Data will be collected from questionnaires, document analyses, semistructured interviews, and structured observations and will cover different aspects of process evaluation. These include examining the context to explore existing patterns, changes in patterns, attitudes, and interactions; analyzing the implementation of intervention elements; and exploring the complex causal pathways and mediators of the intervention. Qualitative data will be analyzed using thematic analysis. The data will then be combined using between-methods triangulation**.**

**Results:**

This project received funding on March 1, 2022. The intervention phase and recruitment for the process evaluation began on March 1, 2023, and the recruitment is expected to end on September 30, 2025. At the time of protocol submission in June 2023, a total of 8 doctors from hematology and oncology practices were enrolled. Data collection began on March 14, 2023.

**Conclusions:**

The *Trans-sectoral Personalised Care Concept for Patients with Rare Cancers* project is a complex intervention that is to be implemented in an equally complex health care context. The process evaluation will help understand the influence of contextual factors and assess the mechanisms of change.

**Trial Registration:**

ISRCTN registry ISRCTN16441179; https://doi.org/10.1186/ISRCTN16441179

**International Registered Report Identifier (IRRID):**

DERR1-10.2196/49731

## Introduction

### Background

Approximately 24% of all emerging cancers are rare cancers, making them cancers with an incidence of <6 in 100,000 people per year. The *rare tumor diseases* category includes almost 200 different entities. Compared with patients with common tumor diseases, patients with rare tumor diseases develop the disease at a younger age, and the overall survival is significantly poorer [1].

Patients in the German health care system can be treated as both inpatients and outpatients in office-based settings. In particular, patients with rare, chronic, or complex diseases often cross the sector boundaries because of their complex treatment plans and the various professions involved. In the German health care system, interface problems between the office-based and inpatient sectors may cause gaps in the delivery of care [2]. Therefore, it is important to improve the design of interfaces and, thus, the coordination and integration of inpatient and office-based health care. It is the physicians’ task to ensure continuous medical treatment during the transition from the inpatient sector to the continuing care sector. In particular, the treatment of patients with extensive care needs requires cooperation between service providers, both in the intersectoral area (office-based and inpatient) and intrasectoral area (eg, between different medical disciplines). The treatment path is associated with a high level of coordination and information needs between colleagues providing cotreatment or further treatment [3] (eg, information about supportive treatment, potential side effects, or complications during hospitalization). Digitalization is essential for the development of transsectoral and cross-disciplinary supply structures. Digital technologies can help improve documentation, communication, and networking in medical care [4].

Patients with cancer have to face complex considerations when making decisions about their own treatment [5] in a challenging life situation. They show a strong preference for shared decision-making (47.1%). Slightly more than one-third (36.3%) of German patients with cancer want to decide alone, and 15.9% want the physician to decide [6]. Despite the benefits of shared decision-making (eg, greater satisfaction with the treatment decision and improvement in adherence to cancer treatment), its implementation in clinical practice has hardly improved over the years [5]. Therefore, it is important to address this issue.

### Trans-Sectoral Personalised Care Concept for Patients With Rare Cancers

The Trans-sectoral Personalised Care Concept for Patients with Rare Cancers (TARGET) project includes targeted digital interventions to optimize transsectoral cooperation and coordination of care to improve patient involvement (including shared decision-making) and the medical care of patients with rare cancers. The intervention will be implemented in the model region of Southern Bavaria (Swabia, Upper Bavaria, and Lower Bavaria), Germany, with a maximum of 45 hematologic and oncologic participating practices and the Comprehensive Cancer Center (CCC) Munich. For the evaluation and implementation of TARGET, the Bavarian Association of Statutory Health Insurance Physicians (KVB) and the Professional Association of Resident Haematologists and Oncologists in Bavaria will invite all office-based oncology and hematology specialists to take part. Only patients who are legally insured by AOK Bavaria, the largest statutory health insurance provider in Bavaria, will be recruited.

Deviating from the registration of the main trial [7], the summative evaluation of TARGET will adopt a prospective parallel group design with patients clustered in practices, comparing the control group (practices in Northern Bavaria) with the intervention group (practices in Southern Bavaria). The protocol of the summative evaluation will be published separately. The primary end point is the coordination of care from the patient’s perspective, which will be measured using the validated German version of the Care Coordination Instrument [8]. The secondary end points include progression-free survival, overall survival, course of therapy, time up to diagnosis, and patient-reported outcomes (health-related quality of life, condition, symptoms, and health literacy). An economic evaluation using retrospective data provided by AOK Bavaria will also be conducted. This protocol describes the process evaluation that will occur alongside the main trial.

### Objective

TARGET consists of a variety of components that aim at different target groups, are offered by different groups of professionals, and concern different aspects of patient care. Therefore, a comprehensive process evaluation following the Medical Research Council guidance for process evaluation of complex interventions [9] is planned (1) to analyze contextual factors and their interactions with the intervention components; (2) to describe the intervention, its causal assumptions, and the implementation process; and (3) to assess the mechanisms of change such as participant responses and mediators. The aims are to assess intervention fidelity and the implementation of transsectoral communication, identify and describe the intended and nonintended effects of the intervention, and explore the barriers to and facilitators of implementation [9].

## Methods

The reporting of this protocol follows the criteria of the SPIRIT (Standard Protocol Items: Recommendations for Interventional Trials) [10] (Multimedia Appendix 1) and the UK *MRC framework for the development and evaluation of complex interventions* [11]. Mixed methods will be applied to assess processes and mechanisms at different levels.

### Ethical Considerations

Ethics approval was obtained from the ethics committee of the Martin Luther University Halle-Wittenberg (MLU; 2023-032). The ethics committee will be informed of any changes in the procedure. All the participants will provide written informed consent. They will be informed of their possibility to terminate their participation at any time. The personal data collected are subject to the obligation of confidentiality and provisions of the General Data Protection Regulation. Participating practices will be reimbursed via the selective contract with AOK Bavaria for the time and effort required to participate in the training sessions, interviews, and virtual tumor boards as well as for patient inclusion and data documentation. The total compensation amount is based on the services actually provided. Patients will not receive any compensation.

### Logic Model

**Figure 1:**
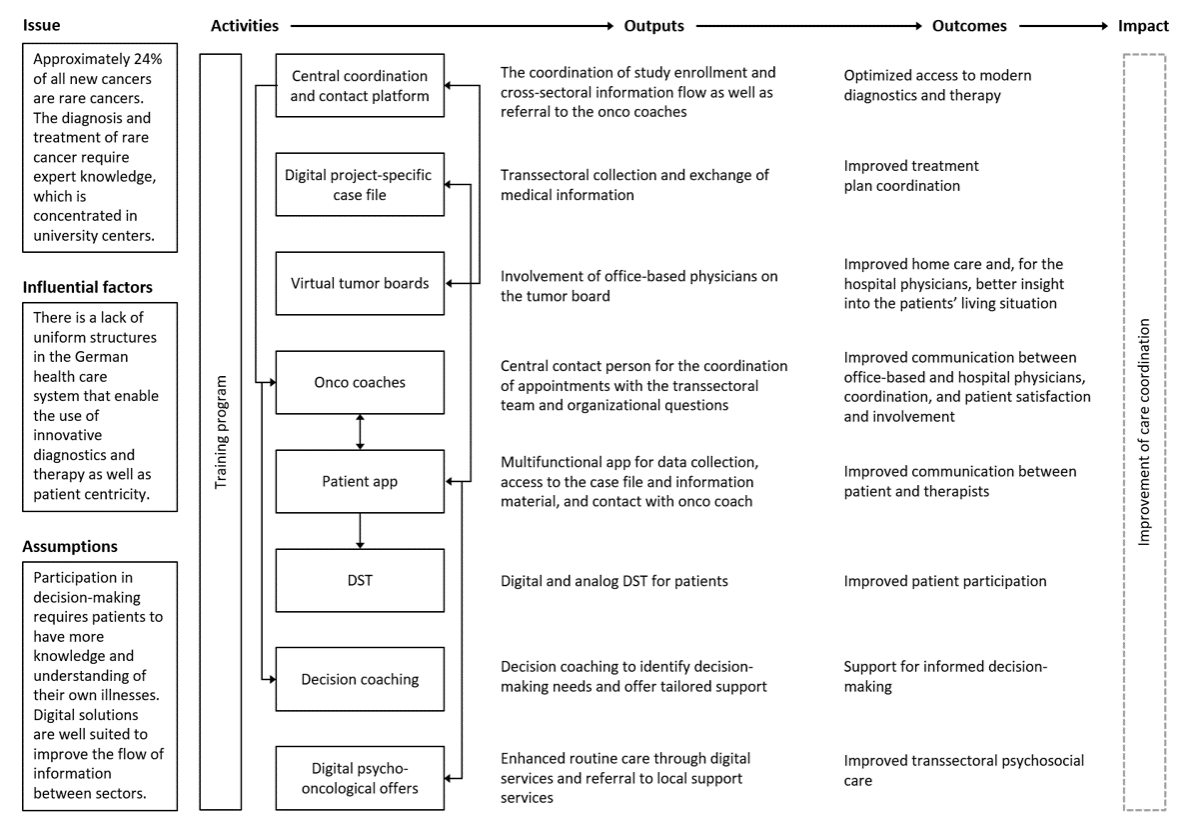
Logic model of the Trans-sectoral Personalised Care Concept for Patients with Rare Cancers (TARGET) intervention. DST: digital support tool.

A logic model was developed for TARGET, along with those responsible for developing the intervention components. This will guide data collection and analysis and is intended to help clarify causal assumptions. The logic model describes the intervention, its components, and its implementation. It also identifies the target group of an intervention component and how it interacts with other components and describes the current context (Figure 1).

### TARGET Intervention and Implementation

TARGET is a complex intervention comprising different components designed to promote cross-sectoral exchange and patient-centered care. The components of the intervention are as follows.

#### Central Coordination and Contact Platform

The participating hematologic and oncologic practices have the opportunity to quickly contact the team at the CCC Munich through phone or email via the central coordination and contact platform. The contact details can be found on the CCC Munich website. The platform will be staffed by a physician throughout the duration of the TARGET project to coordinate the enrollment of patients into the study and guarantee an optimal exchange of information between the different sectors to optimize joint patient care. The data of the patients enrolled in the study will be sent to Onco Coach for appointment scheduling to enable rapid referral to the respective expert team at the CCC Munich. The overall goal of the central coordination and contact platform is to bring the patient to the right expert as quickly as possible (eg, to optimize access to modern [molecular] diagnostics and therapy). It also serves as an interface for office-based hematologists and oncologists.

#### Onco Coach

All enrolled patients are assigned an Onco Coach as a central contact person for health care coordination issues. The Onco Coach play an important role in coordinating appointments with patients within the transsectoral team and mediate participation in training and supportive measures. In addition, the Onco Coach has limited access to the patient app and will reach out to all the patients via the chat function to establish contact with the treatment team. Patients can chat with the Onco Coach regarding organizational questions and problems. As a result, there is a continuous exchange between the Onco Coach and the treatment team.

##### Decision Coaching

Patients experiencing a decisional conflict will be offered decision coaching delivered by a trained Onco Coach or a physician in the hospital. According to the Ottawa Decision Support Framework [12], the decision coach will identify decisional needs, such as inadequate knowledge, unrealistic expectations, or unclear values, and offer tailored support to address these needs (eg, providing information, verifying understanding, or clarifying personal values).

#### Digital Project-Specific Case File

The digital project-specific case file is used for the transsectoral collection and exchange of medical information by establishing a link between the participating hematologic and oncologic practices and the CCC Munich and thus connects all relevant actors. The aim is to enhance coordination regarding the treatment plan. Interoperability is an essential aspect of the case file. The IT project was registered in vesta, the interoperability directory of the German health care system [13]. Availability is ensured using a platform-independent web application. The case file is a CentraXX-associated module from Kairos GmbH, which was expanded to include an oncological module for TARGET. CentraXX is a research portal focusing on the structured recording of content using biobanking and study management modules. An electronic patient record is the central storage unit in CentraXX [14]. It is technically operated by the Ludwig-Maximilians-University (LMU) Hospital Munich and professionally supervised by the CCC Munich. Plausibility checks ensure improved data quality. The web application will be set up in the hematologic and oncologic practices by the IT department of the CCC Munich. In addition, the practices will receive an on-site briefing and a manual on how to use the case file.

#### Patient App

The patient app is a smartphone app that is used for data collection as well as part of the intervention. It is compatible with and based on the project-specific case file with the following three basic functionalities: (1) documentation and use of various integrated forms, (2) customizable view of the coordinated case file, and (3) provision of information and training materials (eg, information on nutrition and exercise for cancer and fatigue, a decision support tool [DST], and other sources of information and support).

The assessment questionnaires can be accessed and completed via the app interface. Patients can also access a summary of the quality of life, functional, and symptom questionnaires, which can be used as personal documentation and for medical consultations. These documents are also made available to the transsectoral treatment team through the project-specific case file. The results of the questionnaires assessing care coordination and health literacy are not visible to the treatment team. This will be communicated to the patients in the study documents and within the questionnaires to avoid social desirability bias. In addition to data collection, the National Comprehensive Cancer Network (NCCN) distress thermometer [15] serves as a screening tool for the provision of psycho-oncology services (see the *Digital Psycho-Oncological Offers* section).

The patient app also allows the Onco Coach to contact the patient. All data are transmitted to the CentraXX department of the LMU Hospital. This integration makes it possible to combine all the recorded parameters and scientifically evaluate the collected data. The app is available for iOS (Apple Inc) and Android (Google LLC) and can be downloaded by patients via a QR code. Patients will receive instructions from their attending physician when they are enrolled in the study.

##### DST for Informed Decision-Making

To prepare for medical consultations, patients will have access to a generic DST [16] that aims at supporting informed decision-making. It is based on the Ottawa Personal Decision Guide and has been complemented with decision-related questions from question prompt lists for patients in oncology [17]. The DST will enable patients to ask for important information about their options regarding treatment or diagnostic procedures and to weigh their preferences for these options. It also includes guidance on how to deal with decisional needs, such as missing information, decisional uncertainty, and a lack of support. The tool will be available as a PDF version and in a web-based format via LimeSurvey (LimeSurvey GmbH). The links to the PDF version and LimeSurvey will be made available via the patient app and a flyer.

#### Virtual Tumor Boards

TARGET involves the establishment of internet-based transsectoral tumor boards, which enable joint decision-making and transsectoral information flow. Internet-based tumor boards have already been established at the CCC Munich and will be made available to office-based hematologists and oncologists as a component of TARGET. Within the tumor boards, office-based hematologists and oncologists and the physicians of the CCC Munich discuss and jointly determine therapeutic paths. Especially in the field of precision oncology, the participating office–based hematologists and oncologists will benefit from the expertise at the CCC Munich, as a molecular tumor board has already been established there. The transsectoral tumor board allows for the sharing of this knowledge across sectors. Thus, close-to-home care can be improved, and the CCC Munich physicians can gain deeper insights into the patient’s living situation. Furthermore, central tumor boards provide optimized access to clinical studies.

#### Digital Psycho-Oncological Offers

Psycho-oncology is standard care in certified centers such as the CCC Munich but not nationwide in outpatient settings. The aim of this intervention component is to improve cross-sectoral psychosocial care delivery and thus overcome urban-rural and outpatient-inpatient differences by ensuring the availability of timely psycho-oncological support.

For patients participating in this study, intensive multiprofessional collaboration, the rapid availability of services, and specific expertise and background knowledge in the psycho-oncological counseling context are required. To initiate psycho-oncological counseling, the NCCN distress thermometer [15] will be provided at the initial visit and every 4 weeks via the patient app. If the score is ≥5 or if the patient requests psycho-oncological support, the patient will be contacted by a psycho-oncologist within the first 24 to 48 hours after distress screening. The patient will also receive contact details. The consultation can take place via telephone, in person at the CCC Munich, or digitally via RED Connect (RED Digital Cinema, LLC) [18] and will last approximately 50 minutes. All existing psycho-oncological support opportunities will be offered to the patients (one-on-one counseling, couple or family consultations, open consultation hours, and group offers for patients and their relatives or caregivers). In addition, patients will receive information about different support offers as well as patient events, and patient chats via the patient app.

Provision of information about and potential referral to local support services will be facilitated through an intensive exchange with the existing networks called Network Psychosocial Oncology Munich, the KVB, the Bavarian Chamber of Psychotherapists, and the Bavarian Cancer Society.

#### Training Program

For the successful implementation of the new care concept, a modular training concept was developed, which will be implemented via the KVB’s e-learning platform, CuraCampus. The training concept includes (1) training on the overall project, (2) training in shared decision-making, and (3) web-based learning after 1 year of the project.

First, the training on the overall project is mandatory for physicians from hematologic and oncologic practices. The asynchronous web-based course will last a maximum of 45 minutes.

Second, the 45-minute asynchronous web-based training on shared decision-making aims to introduce the concept of informed shared decision-making and the use of the DST in consultations. For physicians from hematologic and oncologic practices, this course is also offered on CuraCampus and advertised in mandatory training. Physicians at the hospital will also have the opportunity to take part in the course via a cloud-based platform of the University of Bremen. The offer is advertised via flyers. Teaching methods include a screencast lecture and a best practice video of a simulated physician-patient consultation in which a decision is made about palliative treatment options for pancreatic cancer. In addition, barriers and facilitators for implementing the concept of informed shared decision-making will be discussed. A voluntary multiple-choice knowledge test at the end of the course allows participants to earn continuing medical education credits. Physicians are also invited to participate in a voluntary 2-hour web-based communication training to achieve shared decision-making skills through simulated patient discussions.

Third, after 1 year of the project, another training will be offered as a refresher and update to discuss previous barriers and process adjustments and changes.

In addition, office-based hematologists and oncologists will be trained on how to use the digital case file by the IT team.

The Onco Coach will have the opportunity to participate in a 1-day decision coaching training in a blended learning format. A preexisting training program for decision coaches in oncology [19] was adapted to the special needs of the target group and to decision-making in the field of rare cancer diseases. It focuses on skills in decision coaching, shared decision-making, evidence-based health information, and (risk) communication. Decision coaching skills are trained through simulated conversations based on case vignettes.

### Participants and Recruitment

We will include the following people in this evaluation.

1. We will include patients who participate in the intervention phase. Using theoretical sampling, we will take into account factors such as gender, age, diagnosis, time up to diagnosis, and course of therapy. These patient characteristics and clinical data will be extracted from medical records. The willingness to participate in an interview is requested via the central coordination and contact platform. Over the entire intervention period, a total of approximately 40 interviews are planned. Theoretical data saturation will be the decisive criterion for the number of interviews.
2. We will include all persons and staff involved in the development and implementation of the intervention (Onco Coach, psychologists, physicians on the contact platform, IT staff, and KVB staff)
3. We will include physicians from the LMU Hospital and the hospital of the Technical University Munich (TUM) who are involved in the treatment of patients during the course of the project. Owing to the number of departments in the clinics, we will use a theoretical sample based on the extent of patient contact or contact with the intervention components. At the physician level, a snowball sampling will be used. Recruitment will be done by approaching supervisors via either email or telephone. We estimate the number of interviews over the course of the project to be 10 to 15, taking theoretical data saturation into consideration.
4. We will include participating physicians from the hematologic and oncologic practices. In the preliminary phase, the first interview is conducted with physicians from all the partaking practices. The second interview will take place at different times during the intervention phase. The sampling will be based on the characteristics of the practices and physicians (eg, the size of the practices or experience of the physicians), recruitment rate, and contact with the intervention components (eg, participation in the virtual tumor board). The aim is to map as many different contextual conditions and factors of the intervention as possible. Recruitment will be done via telephone or email.

### Data Collection

#### Overview

Data will be collected using a mixed methods approach at the beginning, during, and at the end of the intervention. Data will be collected through questionnaires, document analyses, semistructured interviews, and structured observations and will cover different aspects of process evaluation.

The personal data collected are subject to the obligation of confidentiality and the provisions of data protection law. The data will be used exclusively for the purpose specified in the consent form. Initially, the data will be pseudonymized, and then they will be anonymized during the later course of the study. After completion of the study, the transcribed audio files will be deleted.

If available, the pseudonyms assigned in the main trial will be used. Patients will be referred for the process evaluation through the established central coordination and contact platform. The key list remains at the LMU. By agreeing to participate in an interview as part of the process evaluation, the patients explicitly consent to being contacted by the staff of the MLU.

All data collected are processed on access-protected computers and then stored safely and securely for 10 years at the Institute of Health and Nursing Science at the MLU. The pseudonymized data will not be passed on to third parties. The publication of the data takes place exclusively in an anonymized form.

All participants will receive information about the study, and informed consent will be obtained (Multimedia Appendix 2). Informed consent will be obtained by a member of the MLU evaluation team, except for patients whose consent will be obtained through the central coordination and contact platform. Interested persons will receive written information about the study project, with the opportunity to ask questions via a conversation. They will then be provided with a consent form to sign. Before data collection begins, there will be another opportunity to ask questions and confirm consent.

After the inclusion of the physicians from the hematologic and oncologic practices, the baseline characteristics are recorded using the web-based survey application LimeSurvey. The link to the survey and an individual access code will be sent to the practices via email. If necessary, an email reminder will also be sent.

Several interviews will be conducted in person, through video conference via Cisco Webex [20], or through telephone. Interview guides were developed for each target group and topic of interest, which will be piloted in the first 3 to 5 interviews and adapted, if necessary. The audio-recorded interviews will be transcribed. There is no plan to send the interview transcripts to the participants. Structured observations will be conducted using a protocol with guiding themes. The protocols will also be piloted during the first structured observations. The objectives of the data collection are described in subsequent sections and summarized in Tables 1 to 3.

**Table 1:** Data collection on the context of the intervention.

Intervention components and participants			Method		Assessments
**Central platform for coordination and contact**					
	Physicians from the coordination platform and office-based hematologists and oncologists	Semistructured interviews		Experiences and facilitating and hindering factors regarding the contact platform	
**Onco Coach**					
	Onco Coach	Semistructured interviews		Baseline data (eg, vocational education and training and professional experience) and understanding of one’s role and expectations	
	Patients, hospital physicians, office-based hematologists and oncologists, and project participants of other intervention components	Semistructured interviews		Experiences and facilitating and hindering factors regarding cooperation	
**Digital project-specific case file**					
	Users (hospital physicians, office-based hematologists and oncologists, and project participants of other intervention components)	Semistructured interviews		Experiences and facilitating and hindering factors regarding the use of the case file	
	Office-based hematologists and oncologists	Questionnaire		Baseline data on the practice and person (location, area covered, connections to clinics or centers, composition and characteristics of the practice team, and IT equipment)	
**Patient app**					
	Patients and users of other intervention components (Onco Coach and psycho-oncologists)	Semistructured interviews		Experiences and facilitating and hindering factors regarding the use of the app	
**Virtual tumor board**					
	Hospital physicians and office-based hematologists and oncologists	Semistructured interviews and structured observation		Existing structures, experiences, and facilitating and hindering factors with regard to participation in and cooperation on the tumor board	
**Decision support tool**					
	Patients, office-based hematologists and oncologists, and hospital physicians	Semistructured interviews		Experiences and facilitating and hindering factors regarding the application	
**Decision coaching**					
	Patients and Onco Coach	Semistructured interviews		Experiences and facilitating and hindering factors regarding the decision coaching	
**Digital psycho-oncological offers**					
	Patients and psycho-oncologists	Semistructured interviews		Existing structures, experiences, and facilitating and hindering factors regarding the services offered	
**Training program**					
	Hospital physicians, office-based hematologists and oncologists, and Onco Coach	Semistructured interviews		Experiences and facilitating and hindering factors regarding the training program	

**Table 2:** Data collection on the implementation process.

Intervention components and participants			Method		Assessments
**Central platform for coordination and contact**					
	Documentation of the central platform for coordination and contact	Document analysis		Number of contacts (medium and topic) and recruitment rate	
	Hospital physicians, Onco Coach, office-based hematologists and oncologists, and physicians from the contact platform	Semistructured interviews		Experiences regarding accessibility, cooperation, and consequences of the contact and changes during the process	
**Onco Coach**					
	Onco coach documentation	Document analysis		Number of contacts with patients, hospital physicians, and office-based hematologists and oncologists and medium and topic of the contact	
	Patients, Onco Coach, and hospital physicians	Semistructured interviews		Experiences regarding accessibility, cooperation, and consequences of the contact and changes during the process	
**Digital project-specific case file**					
	Users	Semistructured interviews		Application of the digital case file and exchange among participants with the help of the case file	
	IT department	Document analysis and semistructured interviews		Support requests and solutions and changes during the process	
**Patient app**					
	Patients and users of other intervention components (Onco Coach and psycho-oncologists)	Semistructured interviews		Use of the app and changes during the process	
	IT department	Document analysis and semistructured interviews		Support requests and solutions and changes during the process	
**Virtual tumor board**					
	Hospital physicians and office-based hematologists and oncologists	Semistructured interviews and structured observation		Tumor board procedure and cooperation between hospital physicians and office-based physicians and participation of office-based hematologists and oncologists	
**Decision support tool**					
	Patients, office-based hematologists and oncologists, and hospital physicians	Semistructured interviews		Use of the decision support tool	
	Decision support tool	Document analysis		Number of times the decision support tool was used and frequency of the topics for which the tool was used	
**Decision coaching**					
	Patients and Onco Coach	Semistructured interviews		Services offered and procedure of the decision coaching	
	Decision coaching documentation	Document analysis		Number of times decision coaching was carried out	
**Digital psycho-oncological offers**					
	Patients and psycho-oncologists	Semistructured interviews		Experiences and facilitating and hindering factors regarding the services offered	
	Documentation of the digital psycho-oncological offers	Document analysis		Cooperation with external psycho-oncologists	
**Training program**					
	Hospital physicians, office-based hematologists and oncologists, and Onco Coach	Semistructured interviews		Satisfaction and practical applicability of the contents	
	Training program documentation	Document analysis		Number of users and evaluation of test results	

**Table 3:** Data collection on the mechanisms of impact.

Intervention components and participants			Method		Assessments
**Central platform for coordination and contact**					
	Physicians from the coordination platform and office-based hematologists and oncologists	Semistructured interviews		Experiences and attitudes regarding the contact platform in terms of care coordination	
**Onco Coach**					
	Patients, hospital physicians, office-based hematologists and oncologists, and project participants of other intervention components	Semistructured interviews		Experiences and attitudes regarding the services supplied by the Onco Coach in terms of care coordination	
**Digital project-specific case file**					
	Users (hospital physicians, office-based hematologists and oncologists, and project participants of other intervention components)	Semistructured interviews		Experiences and attitudes regarding the case file in terms of care	
**Patient app**					
	Patients and users of other intervention components (Onco Coach and psycho-oncologists)	Semistructured interviews		Experiences and attitudes regarding the app in terms of care	
**Virtual tumor board**					
	Hospital physicians and office-based hematologists and oncologists	Semistructured interviews		Experiences and attitudes regarding the tumor board in terms of care	
**Decision support tool**					
	Patients, office-based hematologists and oncologists, and hospital physicians	Semistructured interviews		Experiences and attitudes regarding the decision guide in terms of care	
**Decision coaching**					
	Patients and Onco Coach	Semistructured interviews		Experiences and attitudes regarding the decision coaching in terms of care	
**Digital psycho-oncological offers**					
	Patients and psycho-oncologists	Semistructured interviews		Experiences and attitudes regarding the services offered in terms of care	
**Training program**					
	Hospital physicians, office-based hematologists and oncologists, and Onco Coach	Semistructured interviews		Experiences and attitudes regarding the contents in terms of care	

#### Context of the Intervention

To better understand the mechanisms of change and identify barriers and facilitating factors for implementation, contextual factors will be examined. For this purpose, we will conduct semistructured interviews with responsible persons of the CCC Munich at different points during the intervention phase to explore existing structures, changes in structures, attitudes, and mutual influences. In addition, we will conduct semistructured interviews with the responsible persons, staff, and stakeholders of the intervention components and collect descriptive data on the existing resources. To better understand and describe the context in which hematologic and oncologic practices are embedded, we will also conduct semistructured interviews during the intervention phase. Baseline characteristics such as the location of the practice, practice structures, connections or cooperation with a clinic or center, and IT equipment will be assessed.

#### Implementation Process

We will document the implementation process of the intervention, success and failure of recruitment, and participation rates. To answer the question of how the intervention components were implemented, we will conduct semistructured interviews with all persons involved in the intervention (patients, physicians in hematologic and oncologic practices, and staff of the CCC Munich). Furthermore, we will address the question of whether individual intervention components were implemented as intended. This includes the fidelity of the intervention, the degree of implementation, conscious and unconscious adjustments, and the extent to which the respective components reached the target group. For this purpose, we will conduct quantitative and qualitative document analyses on different issues (eg, the number of participants in the training courses, the frequency of psycho-oncological consultations, the completeness of documentation, the use of the DST, and IT support). We will also use structured observations for individual intervention components (eg, the tumor board).

#### Mechanisms of Impact

To understand the impact of the intervention components on care coordination, we will conduct semistructured interviews with patients in the intervention group, physicians in hematology and oncology practices, persons and staff responsible for the intervention components, and physicians in the LMU and TUM. The aim is to understand the complex causal pathways and mediators of the intervention. The theoretical concept of care coordination will be the basis for the exploration. In particular, the focus is on the domains of communication and information (including all processes, communication, and information within the attending practice) and needs-based navigation (need-assessment and needs-based navigation outside the caregiver’s institution, including all stakeholders in the health care process). Furthermore, spontaneously reported unintended effects of both the intervention components and the process evaluation itself will be collected. Significant influences will be discussed with the project team.

### Data Analysis

Analyses of the baseline characteristics will be descriptive. The quantitative data will be evaluated through descriptive analysis. Qualitative data will be collected and analyzed in an iterative process until data saturation is reached through thematic analysis, according to Braun and Clarke [21]. Data analysis will be carried out by 2 people independently. In case of disagreement, consensus will be reached through discussion with a third person. Finally, the data will be combined using between-methods triangulation [22], the individual components of the process evaluation will be brought together, and configurations that have led to a high degree of cross-sectoral coordinated care will be identified at the practice level. The results of the triangulation will be analyzed in reflection talks after the completion of the study. The reflection talks serve to ensure sustainability, exchange experiences, and jointly evaluate the results. The processes of data collection and analysis are iterative until data saturation is assumed. The results of the interviews will be sent to all the participants via email.

## Results

The project received funding on March 1, 2022. The intervention phase and recruitment for the process evaluation began on March 1, 2023, and the recruitment is expected to end on September 30, 2025. At the time of protocol submission in June 2023, a total of 8 doctors from hematology and oncology practices were enrolled. Data collection began on March 14, 2023.

## Discussion

### Overview

The TARGET project is a complex intervention that is to be implemented in an equally complex health care context. Whether the implementation is successful and the intervention achieves its desired effect depends on various factors and their interactions, for example, the contextual factors in the outpatient and clinical settings, the behavior and attitudes of the different stakeholders involved, and the intervention components in relation to routine care. A comprehensive analysis of processes and an assessment of diverse perspectives have been planned alongside a logic model. The logic model will guide data collection and analyses and is intended to help clarify causal assumptions. The findings of the primary outcome *coordination of care* from the patient’s perspective, measured using the validated German version of the Care Coordination Instrument [8], will be accompanied by in-depth interviews with patients and perspectives of physicians. The results will finally be reported according to the Template for Intervention Description and Replication (TIDieR) checklist [23].

The summative evaluation study will follow a parallel group design, and eligible patients will be a heterogeneous group in terms of diagnosis, stage of disease, and course of treatment. Therefore, a comprehensive process evaluation is all the more important to understand the influence of contextual factors, assess the mechanisms of change, identify intended and nonintended effects, and explore the barriers to and facilitators of implementation.

As the evaluators who conduct the process evaluation are an external team from the MLU, it is important to establish a good working relationship with all the stakeholders involved in the intervention development and implementation to ensure a close observation of the intervention. Maintaining sufficient independence while observing the work of the different stakeholders is crucial. Another challenging question is whether the emerging findings should be communicated to the stakeholders. The evaluators can either act as passive observers reporting the results at the end of the project or help correct implementation problems while they occur [9]. We will not engage in continuous quality improvement activities because this might compromise the external validity of the evaluation [9], although serious observations will be discussed with the project team.

### Limitations

This process evaluation also has limitations. Even after intensive exchange with the developers of the intervention, some ambiguity over the content of the intervention, its components, and how it is intended to work remains. Owing to the high complexity, it is hardly possible to record all processes and data, so some uncertainties will remain. For example, no observation or analysis of consultations is planned. Furthermore, it will be difficult to find out the reasons for nonparticipation, both for physicians and patients, because there will be no direct access to the group. However, it is better to answer the most important core questions well than to try to answer too many questions [9]. Therefore, we started to list causal assumptions within a logic model. Moreover, additional questions may arise during the course of the process evaluation [9]. Overall, it should be considered that access to the participants and data depends on the readiness and feasibility for it in an overburdened health care system.

### Conclusions

The results and detailed descriptions of the process evaluation will help interpret the results of the TARGET intervention and put them into the correct context. In addition, the results may inform future projects that adapt TARGET or its individual components to different fields of cancer care or beyond. The results may also raise needs regarding the enhancement of the skills of the involved professions.

## Appendix

Multimedia Appendix 1 SPIRIT (Standard Protocol Items: Recommendations for Interventional Trials) checklist.

Multimedia Appendix 2 Model informed consent form.

## Abbreviations

CCC: Comprehensive Cancer Center
DST: decision support tool
KVB: Bavarian Association of Statutory Health Insurance Physicians
LMU: Ludwig-Maximilians-University
MLU: Martin Luther University Halle-Wittenberg
NCCN: National Comprehensive Cancer Network
SPIRIT: Standard Protocol Items: Recommendations for Interventional Trials
TARGET: Trans-sectoral Personalised Care Concept for Patients with Rare Cancers
TIDieR: Template for Intervention Description and Replication
TUM: Technical University Munich

## Data Availability

The data sets generated and analyzed during this study are available upon request from AS.
