# Supplementary material for: Enhancing Transsectoral Interdisciplinary Patient-Centered Care for Patients With Rare Cancers: Protocol for a Mixed Methods Process Evaluation
Source: JMIR Res Protoc. 2023 Oct 12;12:e49731. doi: 10.2196/49731 (PMC10603554; doi:10.2196/49731)
Supplement: Multimedia Appendix 1 [file resprot_v12i1e49731_app1.pdf]

SPIRIT Checklist [10]

		Reporting Item	Page and Line Number	Reason if not applicable
**Administrative information**				
Title	#1	Descriptive title identifying the study design, population, interventions, and, if applicable, trial acronym	Page 1; Line 2-4	
Trial registration	#2a	Trial identifier and registry name. If not yet registered, name of intended registry	Page 4; Line 80-81	Since there is no registration for process evaluations, we have indicated registration for the overall project.
Trial registration: data set	#2b	All items from the World Health Organization Trial Registration Data Set	n/a	As the registration refers to the whole project, the data given would not coincide with this protocol. We have therefore abstained from doing so.
Protocol version	#3	Date and version identifier	Page 20 Line 475-478	
Funding	#4	Sources and types of financial, material, and other support	Page 20; Line 480-487	
Roles and responsibilities: contributorship	#5a	Names, affiliations, and roles of protocol contributors	Page 1-2; Line 5-40<br><br>Page 20; 492-494	
Roles and responsibilities: sponsor contact information	#5b	Name and contact information for the trial sponsor	n/a	Information about the sponsor is already included in the study registration of the main study.

Roles and responsibilities: sponsor and funder	#5c	Role of study sponsor and funders, if any, in study design; collection, management, analysis, and interpretation of data; writing of the report; and the decision to submit the report for publication, including whether they will have ultimate authority over any of these activities	Page 20; Line 482-484	
Roles and responsibilities: committees	#5d	Composition, roles, and responsibilities of the coordinating centre, steering committee, endpoint adjudication committee, data management team, and other individuals or groups overseeing the trial, if applicable (see Item 21a for data monitoring committee)	n/a	There are no committees or groups involved in the coordination and implementation of the study.
**Introduction**				
Background and rationale	#6a	Description of research question and justification for undertaking the trial, including summary of relevant studies (published and unpublished) examining benefits and harms for each intervention	Page 4-6; Line 85-135	
Background and rationale: choice of comparators	#6b	Explanation for choice of comparators	n/a	The process evaluation does not contain a control intervention.
Objectives	#7	Specific objectives or hypotheses	Page 6; Line 137-146	
Trial design	#8	Description of trial design including type of trial (eg, parallel group, crossover, factorial, single group), allocation ratio, and framework (eg,	Page 7; Line 150-151	

		superiority, equivalence, non-inferiority, exploratory)		
**Methods: Participants, interventions, and outcomes**				
Study setting	#9	Description of study settings (eg, community clinic, academic hospital) and list of countries where data will be collected. Reference to where list of study sites can be obtained	Page 5; Line 112-135	
Eligibility criteria	#10	Inclusion and exclusion criteria for participants. If applicable, eligibility criteria for study centres and individuals who will perform the interventions (eg, surgeons, psychotherapists)	Page 14-15; Line 311-336	
Interventions: description	#11a	Interventions for each group with sufficient detail to allow replication, including how and when they will be administered	Page 8-14; Line 171-310	
Interventions: modifications	#11b	Criteria for discontinuing or modifying allocated interventions for a given trial participant (eg, drug dose change in response to harms, participant request, or improving / worsening disease)	n/a	The process evaluation accompanies the main study. Criteria are defined in the main study.
Interventions: adherance	#11c	Strategies to improve adherence to intervention protocols, and any procedures for monitoring adherence (eg, drug tablet return; laboratory tests)	n/a	The process evaluation accompanies the main study and describes the implementation process of the TARGET intervention without directly influencing it.
Interventions: concomitant care	#11d	Relevant concomitant care and interventions that are permitted or prohibited during the trial	n/a	The process evaluation accompanies the main study and describes the implementation process of the TARGET

				intervention without directly influencing it.
Outcomes	#12	Primary, secondary, and other outcomes, including the specific measurement variable (eg, systolic blood pressure), analysis metric (eg, change from baseline, final value, time to event), method of aggregation (eg, median, proportion), and time point for each outcome. Explanation of the clinical relevance of chosen efficacy and harm outcomes is strongly recommended	Page 16-18; Line 375-411<br><br>Table 1	
Participant timeline	#13	Time schedule of enrolment, interventions (including any run-ins and washouts), assessments, and visits for participants. A schematic diagram is highly recommended (see Figure)	n/a	The process evaluation, with the exception of a baseline survey for resident doctors, includes an accompanying evaluation throughout the intervention period. Due to the scope of the intervention components but still the same assessment process, we do not believe that a diagram would be a useful addition to the description in Page 14 Line 292-296 and table 1. A schematic diagram will be included in the study protocol of the main study.
Sample size	#14	Estimated number of participants needed to achieve study objectives and how it was determined, including clinical and statistical assumptions supporting any sample size calculations	Page 14-15; Line 312-336	

Recruitment	#15	Strategies for achieving adequate participant enrolment to reach target sample size	Page 14-15; Line 312-336	
**Methods: Assignment of interventions (for controlled trials)**				
Allocation: sequence generation	#16a	Method of generating the allocation sequence (eg, computer-generated random numbers), and list of any factors for stratification. To reduce predictability of a random sequence, details of any planned restriction (eg, blocking) should be provided in a separate document that is unavailable to those who enrol participants or assign interventions	n/a	The reported study is not a controlled trial.
Allocation concealment mechanism	#16b	Mechanism of implementing the allocation sequence (eg, central telephone; sequentially numbered, opaque, sealed envelopes), describing any steps to conceal the sequence until interventions are assigned	n/a	The reported study is not a controlled trial.
Allocation: implementation	#16c	Who will generate the allocation sequence, who will enrol participants, and who will assign participants to interventions	n/a	The reported study is not a controlled trial.
Blinding (masking)	#17a	Who will be blinded after assignment to interventions (eg, trial participants, care providers, outcome assessors, data analysts), and how	n/a	The reported study is not a controlled trial.
Blinding (masking): emergency unblinding	#17b	If blinded, circumstances under which unblinding is permissible, and procedure for revealing a	n/a	The reported study is not a controlled trial.

		participant's allocated intervention during the trial		
**Methods: Data collection, management, and analysis**				
Data collection plan	#18a	Plans for assessment and collection of outcome, baseline, and other trial data, including any related processes to promote data quality (eg, duplicate measurements, training of assessors) and a description of study instruments (eg, questionnaires, laboratory tests) along with their reliability and validity, if known. Reference to where data collection forms can be found, if not in the protocol	Page 15-16; Line 338-373<br>Table 1	
Data collection plan: retention	#18b	Plans to promote participant retention and complete follow-up, including list of any outcome data to be collected for participants who discontinue or deviate from intervention protocols	n/a	The process evaluation concerns only a short period of time and one-time contact for data collection. The general plans will be described in the protocol of the main trial.
Data management	#19	Plans for data entry, coding, security, and storage, including any related processes to promote data quality (eg, double data entry; range checks for data values). Reference to where details of data management procedures can be found, if not in the protocol	Page 18; Line 416-417	
Statistics: outcomes	#20a	Statistical methods for analysing primary and secondary outcomes. Reference to where other details of the statistical analysis plan can be found, if not in the protocol	Page 18; Line 413-416	Statistical methods for analysing primary and secondary outcomes will be described in the protocol of the main trial. Data

				analysis of the process evaluation is described in this protocol.
Statistics: additional analyses	#20b	Methods for any additional analyses (eg, subgroup and adjusted analyses)	n/a	Will be described in the protocol of the main trial. In this process evaluation, quantitative data will be analysed in a descriptive way only.
Statistics: analysis population and missing data	#20c	Definition of analysis population relating to protocol non-adherence (eg, as randomised analysis), and any statistical methods to handle missing data (eg, multiple imputation)	n/a	Will be described in the protocol of the main trial. In this process evaluation, quantitative data will be analysed in a descriptive way only.
**Methods: Monitoring**				
Data monitoring: formal committee	#21a	Composition of data monitoring committee (DMC); summary of its role and reporting structure; statement of whether it is independent from the sponsor and competing interests; and reference to where further details about its charter can be found, if not in the protocol. Alternatively, an explanation of why a DMC is not needed	n/a	Due to the minimal risk of the main study and this process evaluation, no data monitoring committee is established.
Data monitoring: interim analysis	#21b	Description of any interim analyses and stopping guidelines, including who will have access to these interim results and make the final decision to terminate the trial	n/a	Due to the minimal risk of the main study and this process evaluation, no interim analyses and sopping guidelines are established.
Harms	#22	Plans for collecting, assessing, reporting, and managing solicited and spontaneously reported adverse events and other unintended effects of trial interventions or trial conduct	Page 18; Line 409-411	

Auditing	#23	Frequency and procedures for auditing trial conduct, if any, and whether the process will be independent from investigators and the sponsor	n/a	The audit concerns the main study and not the process evaluation.
**Ethics and dissemination**				
Research ethics approval	#24	Plans for seeking research ethics committee / institutional review board (REC / IRB) approval	Page 20; Line 463-468	
Protocol amendments	#25	Plans for communicating important protocol modifications (eg, changes to eligibility criteria, outcomes, analyses) to relevant parties (eg, investigators, REC / IRBs, trial participants, trial registries, journals, regulators)	Page 20: Line 465-466	
Consent or assent	#26a	Who will obtain informed consent or assent from potential trial participants or authorised surrogates, and how (see Item 32)	Page 14; Line 324-326	
Consent or assent: ancillary studies	#26b	Additional consent provisions for collection and use of participant data and biological specimens in ancillary studies, if applicable	n/a	No ancillary studies will be conducted.
Confidentiality	#27	How personal information about potential and enrolled participants will be collected, shared, and maintained in order to protect confidentiality before, during, and after the trial	Page 14; Line 318-324	
Declaration of interests	#28	Financial and other competing interests for principal investigators for the overall trial and each study site	Page 21; Line 476-477	

Data access	#29	Statement of who will have access to the final trial dataset, and disclosure of contractual agreements that limit such access for investigators	Page 14; Line 327-330	
Ancillary and post trial care	#30	Provisions, if any, for ancillary and post-trial care, and for compensation to those who suffer harm from trial participation	n/a	Will be described in the protocol of the main trial.
Dissemination policy: trial results	#31a	Plans for investigators and sponsor to communicate trial results to participants, healthcare professionals, the public, and other relevant groups (eg, via publication, reporting in results databases, or other data sharing arrangements), including any publication restrictions	n/a	Will be described in the protocol of the main trial.
Dissemination policy: authorship	#31b	Authorship eligibility guidelines and any intended use of professional writers	n/a	Will be described in the protocol of the main trial.
Dissemination policy: reproducible research	#31c	Plans, if any, for granting public access to the full protocol, participant-level dataset, and statistical code	Page 20; Line 469-471	Further details will be described in the protocol of the main trial.
**Appendices**				
Informed consent materials	#32	Model consent form and other related documentation given to participants and authorised surrogates	Additional file 2	

Biological specimens	#33	Plans for collection, laboratory evaluation, and storage of biological specimens for genetic or molecular analysis in the current trial and for future use in ancillary studies, if applicable	n/a	The procedures described are not planned in the process evaluation.
