# Supplementary material for: Enhancing Transsectoral Interdisciplinary Patient-Centered Care for Patients With Rare Cancers: Protocol for a Mixed Methods Process Evaluation
Source: JMIR Res Protoc. 2023 Oct 12;12:e49731. doi: 10.2196/49731 (PMC10603554; doi:10.2196/49731)
Supplement: Multimedia Appendix 2 [file resprot_v12i1e49731_app2.pdf]

CCC MÜNCHEN COMPREHENSIVE CANCER CENTER

Medizinische Fakultät der Martin-Luther-Universität Halle-Wittenberg

Institute for Health and Nursing Science
Magdeburger Straße 8
06112 Halle (Saale)

Informed Consent

For the study: "**Process evaluation of a complex intervention to achieve trans-sectoral interdisciplinary patients-centred care for patients with rare cancers**"

I was fully informed about the aims and procedure of the study by a responsible person within the project and had sufficient time to reach a decision. I was able to ask questions, I understood the replies and also accept these. **I have received information according to the Data Protection Regulations and have been informed of my rights (DS-GVO, Art. 13 – 21).** I know that study participation is voluntary and that I can revoke my consent at any time without giving reasons and that I can have my data deleted (until my data is anonymised) without this decision having any adverse effect on me.

> I have been informed that I can revoke my consent at any time without giving reasons, that in that event my collected data can be deleted until it is anonymised and that the publication of the study results cannot be linked to my person.

I herewith consent to being contacted by staff members of the Martin Luther University Halle-Wittenberg.

I herewith consent to the collection of the following data for the purpose of the above-mentioned study:

- **Recording and analysis of interviews**

I herewith consent in particular to health data (diagnosis, diagnostic, therapies) being thematised and collected for the purpose of the above-mentioned study.

I have no costs or financial benefit from participating in this study.

I know that I am entitled to receive a copy of this informed consent. I herewith declare my voluntary participation in this study.

____________ ____________________________
Place and date | Signature of the study participant

____________ ____________________________
Place and date | Signature of the person giving information about the study

Contact: **Prof. Dr. phil. Anke Steckelberg**
Tel. 0345 557-4106 / 0345 557-1220, E-Mail: anke.steckelberg@medizin.uni-halle.de
